# Beyond the Basics: Unraveling the Complexity of Coronary Artery Calcification

**DOI:** 10.3390/cells12242822

**Published:** 2023-12-12

**Authors:** Satwat Hashmi, Pashmina Wiqar Shah, Zouhair Aherrahrou, Elena Aikawa, Rédouane Aherrahrou

**Affiliations:** 1 Department of Biological and Biomedical Sciences, Aga Khan University, Karachi 74800, Pakistan; satwat.hashmi@aku.edu; 2 Institute for Cardiogenetics, Universität zu Lübeck, 23562 Lübeck, Germany; pashmina.shah@student.uni-luebeck.de (P.W.S.); zouhair.aherrahrou@uni-luebeck.de (Z.A.); 3 DZHK (German Centre for Cardiovascular Research), Partner Site Hamburg/Kiel/Lübeck, University Heart Centre Lübeck, 23562 Lübeck, Germany; 4 Cardiovascular Medicine, Brigham and Women’s Hospital, Harvard Medical School, Boston, MA 02115, USA; eaikawa@bwh.harvard.edu; 5 A.I. Virtanen Institute for Molecular Sciences, University of Eastern Finland, FI-70211 Kuopio, Finland

**Keywords:** coronary artery calcification, atherosclerosis, coronary artery disease, genome-wide association studies, genetics

## Abstract

Coronary artery calcification (CAC) is mainly associated with coronary atherosclerosis, which is an indicator of coronary artery disease (CAD). CAC refers to the accumulation of calcium phosphate deposits, classified as micro- or macrocalcifications, that lead to the hardening and narrowing of the coronary arteries. CAC is a strong predictor of future cardiovascular events, such as myocardial infarction and sudden death. Our narrative review focuses on the pathophysiology of CAC, exploring its link to plaque vulnerability, genetic factors, and how race and sex can affect the condition. We also examined the connection between the gut microbiome and CAC, and the impact of genetic variants on the cellular processes involved in vascular calcification and atherogenesis. We aimed to thoroughly analyze the existing literature to improve our understanding of CAC and its potential clinical and therapeutic implications.

## 1. Introduction

The presence of calcium phosphate deposits in the coronary arteries, known as coronary artery calcification (CAC), indicates the existence of coronary artery disease (CAD). This is especially true for incidental micro- or macrocalcification, which is strongly associated with atherosclerotic burden [1].

Coronary calcium can be detected and quantified using many imaging modalities like intravascular ultrasound, optical coherence tomography, and coronary angiography, but the most common non-invasive method for detecting CAC is computed tomography (CT). CAC scores can be calculated from CT by quantifying the amount of calcified plaque. The Agatston scoring method is considered the gold standard for calculating CAC scores. It involves summing up the scores of all calcified lesions while considering the total calcified area and maximum density of the calcification [2,3].

CAC is independently associated with atherosclerotic disease risk and disease progression [4,5,6]. It has emerged as a strong predictor of future cardiac events [7,8,9]. A prospective multi-ethnic cohort study of atherosclerosis (MESA) showed that CAC was associated with a 10-year risk of future cardiac events [10]. Even in individuals without established CAD, CAC was predictive of cardiac events. A meta-analysis of 45 studies with 192,080 asymptomatic and 32,477 symptomatic patients followed for up to 11 years showed that CAC was associated with increased risk of major adverse cardiovascular and cerebrovascular events (MACE) as well as all-cause mortality [11,12]. In the Mediators of Atherosclerosis in South Asians Living in America (MASALA) study, the 10-year atherosclerotic cardiovascular disease risk in South Asians correlates with the CAC burden [13].

Even a small amount of CAC (CAC score of 1 to 10) can increase the risk of experiencing a cardiovascular event by three times compared with the absence of CAC (CAC score of 0) [14]. In addition, there is a close correlation between the progression of CAC scores and an increasing risk of cardiovascular disease [15]. It has been shown that the risk of a coronary event increased by a factor of 7.7 among patients with a CAC score between 101 and 300 and by a factor of 9.67 among patients with CAC >300 compared with those with a CAC score of 0 [16]. It is still not clear whether the presence of CAC or its progression is more vital for predicting the risk and outcomes [17].

To improve clinical risk prediction and guide the initiation of pharmacological therapy, guidelines recommend performing CAC assessments in patients with borderline to intermediate cardiovascular disease risk (10-year risk between 5 and 20%) [18,19,20]. CAC assessment can also improve therapy allocation criteria in patients without clinical atherosclerotic cardiovascular diseases [21,22].

Several genetic loci associated with CAC have been identified through genome-wide association studies (GWASs) [23,24]. These genetic variants can impact the expression and function of genes involved in vascular calcification, inflammatory responses, and lipid metabolism, contributing to an individual’s overall genetic predisposition to CAC. By understanding the genetic underpinnings of CAC, we can gain insights into the molecular mechanisms underlying its pathogenesis and develop personalized prevention and treatment strategies.

**Figure 1:**
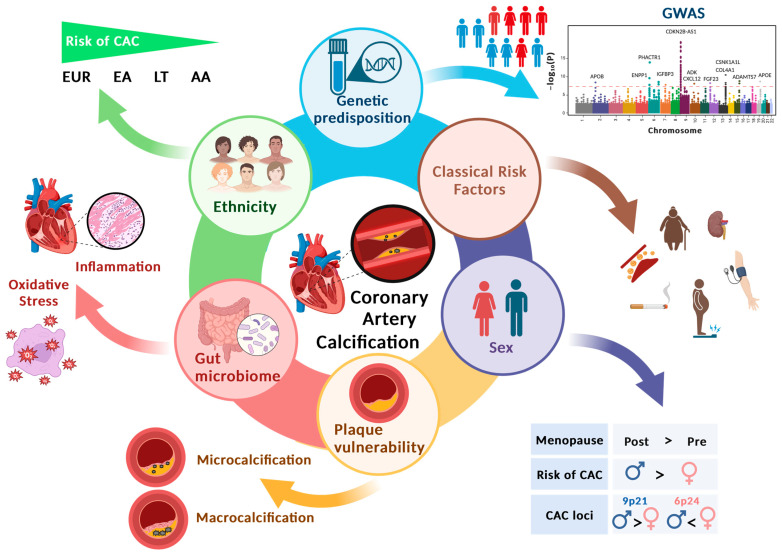
Coronary artery calcification (CAC) and its association with divergent variables/factors. Genetic predisposition of *APOB*, *ENPP1*, *PHACTR1*, *IGFBP3*, *CDKN2B-AS1*, *ADK*, *FGF23*, *CXCL12*, *COL4A1*, *CSNK1A1L*, *ADAMTS7*, and *APOE* have been found to be associated with increased risk of CAC [23,27,28,29,30,31,32,33] (Table 1) along with the classical risk factors: smoking, obesity, old age, high blood pressure, and hyperlipidemia. CAC development is affected by sex; males have a higher probability of CAC than females [1]. The *9p21* locus has a more significant association with CAC in males than females, whereas *6p24* is more strongly associated with females than males [29]. Postmenopausal women are more likely to develop CAC than premenopausal women [34,35]. Regarding ethnicity, the risk for developing CAC is higher in White (EUR), followed by East Asian (Chinese), Latino (LT), and African American individual (AA) [36]. Plaque vulnerability [37,38] and the gut microbiome [39,40] also play a vital role in the pathophysiology of CAC. This figure was created using BioRender.

In this narrative review, we comprehensively examined the current knowledge on the pathophysiology of CAC, specifically regarding its connection to plaque vulnerability, genetic predisposition, and how race and sex can impact CAC. We also reviewed the scientific literature regarding the functional effects of genetic variants on the cellular processes involved in vascular calcification and atherogenesis and explored the association of CAC with microbiome, a newer area of research (Figure 1).

For this review, we conducted a thorough literature search on PubMed and Google Scholar, identifying relevant studies in both human subjects and animals. Our search was limited to English-language research and review articles published up to October 2023. Additionally, we obtained genetic associations from the GWAS catalog [25] and expression quantitative trait loci (eQTL) data were obtained from Genotype-Tissue Expression (GTEx) portal [26].

## 2. Pathophysiology of CAC

Physiologically, calcification occurs in normal developmental processes, which are essential for the integrity and function of the body, such as bones, teeth, and cartilage. However, all kinds of calcium phosphate deposits outside these tissues, including valvular and vascular calcification, are considered pathologic.

Valvular and vascular calcifications may share similar pathogenesis, as similar risk factors have been reported between CAD and valve calcification [41], but only a modest correlation has been shown between CAC score and aortic and mitral valve calcium scores [41,42]. Aortic valve calcification occurs in phases. The first phase is associated with hyperlipidemia and inflammation, evidenced by the presence of oxidized LDL and inflammatory cells. The second phase is related to calcium deposition and ossification, evidenced by the presence of calcium deposits surrounded by osteoblast cells. Low CAC scores correspond to the early aortic valve calcification, while no correlation has been found between aortic valve and high CAC scores [41,43].

Vascular calcification is an organized, regulated cellular process with distinct patterns and mechanisms depending on its location, i.e., intimal or medial calcification. Intimal calcification is the type mostly seen in the coronary arteries [3] and is linked to a systemic process influenced by CAD risk factors, local oxidative stress, and inflammatory mechanisms. On the other hand, medial vascular calcification usually occurs in peripheral arteries of the lower extremities and is viewed as a hydroxyapatite mineralization process within the medial vascular layer [44]. Medial calcification is associated with renal failure and duration of dialysis, hypercalcemia, hyperphosphatemia, diabetes mellitus, aging, and parathyroid hormone abnormalities [3].

Certain blood vessels, particularly the internal mammary artery, seem to be resistant to calcification due to the unique anatomical and functional features of its intimal layer that protect it against the development of atherosclerosis, making it the vessel of choice for coronary artery bypass grafting [45,46].

The first sign of CAC is the presence of microcalcifications, which are small calcified deposits, ranging from 0.5 to 15 um, found in the thickening of coronary artery lesions [47,48,49]. These initial lesions are only histologically seen by special staining for calcium [3].

**Figure 2:**
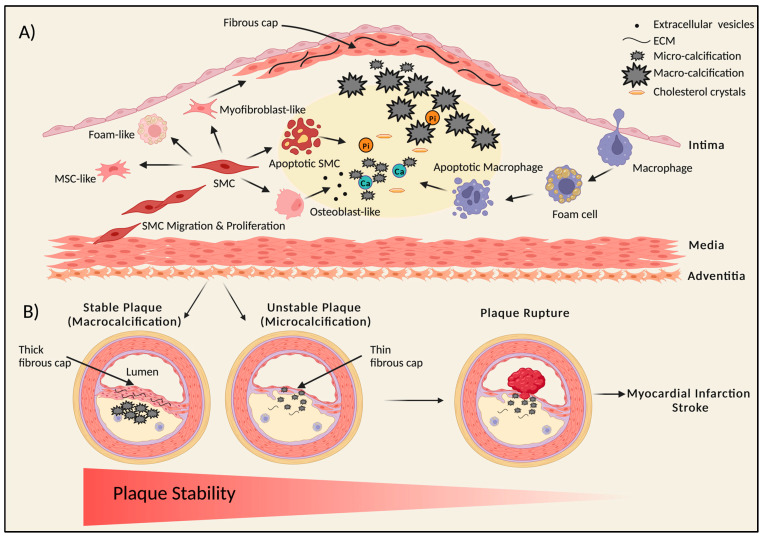
Schematic representation of plaque initiation, calcification, and progression to atherosclerosis. (**A**) Initiation and progression of the atherosclerotic plaque and calcification. During atherosclerosis, vascular smooth muscle cells (SMCs) proliferate and migrate to form the fibrous cap stabilizing the plaque. SMCs can give rise to divergent types of cells via transdifferentiation, like osteoblast-like, myofibroblast-like, foam-like, and mesenchymal-stem-like cells within the plaque core. Release of calcifying extracellular vesicles and apoptosis of SMCs leads to the formation of small, calcified deposits called microcalcifications. Monocytes migrate into the intimal thickening via the endothelium, consume the lipids, and mature into foam cells, which can die and also release extracellular vesicles and apoptotic bodies, thus adding to the calcification process. After microcalcification, larger speckles of calcium punctate deposits are formed, causing macrocalcification, which may progress to calcified sheets and plates. These calcified sheets may fracture, leading to nodular calcification, which leads to plaque rupture and thrombosis. Presence of macrocalcifications in the thin fibrous cap can also contribute to plaque rupture. (**B**) Link between plaque stability and calcification. Stable plaque versus unstable plaque—macrocalcification may lead to stable calcified plaques with a thick collagen-rich extracellular matrix fibrous cap, whereas an unstable plaque has microcalcification with a thin fibrous cap, which is associated with an increased risk of plaque rupture. Microcalcification promotes mechanical stress in the fibrous cap, increasing the propensity to rupture, leading to myocardial infarction or stroke. This figure was created using BioRender.

The microcalcifications in the lipid pool are believed to be caused by the release of calcifying extracellular vesicles [50] or the apoptotic death of smooth muscle cells (SMCs) [1] or macrophages [51,52]. SMCs release round particles called extracellular vesicles, which are approximately 200–300 nanometers in diameter, recruited via sortilin [53]; they contain tissue-nonspecific alkaline phosphatase (TNAP), an enzyme needed for mineralization [54]. In addition, macrophages infiltrate the lipid pool and release their own extracellular vesicles, contributing to the process of microcalcification. These vesicles act as a catalyst for nucleating calcium phosphate crystals associated with a necrotic core containing lipids, macrophages, and apoptotic cells [51] (Figure 2A). Fine microcalcification is likely associated with SMC apoptosis, while larger punctate deposits result from macrophage apoptosis, as evidenced by histological studies [3]. These initial calcifications can only be observed by electron microscopy [3,55]. Although vascular wall cells, including SMCs and macrophages, release extracellular vesicles under normal physiological conditions, these are protected from mineralization by the presence of calcification inhibitors [56].

Over time, extracellular vesicles form macrocalcifications which then coalesce into larger masses and develop macrocalcification in the form of speckles and fragments [3,57]. This will further develop into calcified plaques with calcified sheets or plates. The traditional CT scan resolution is 0.5–1 mm and can detect a lesion with an area of ≥1 mm^2^ [58].

The calcification progresses from the outer rim of the necrotic core into the collagenous matrix around it. The central necrotic core may or may not become calcified [3]. Calcified sheets may fracture, a phenomenon not completely understood [3], giving rise to protuberant nodular calcification. This may lead to the disruption of the endothelial lining and underlying collagen matrix and subsequently acute coronary thrombosis [59]. In addition, recent computational studies have suggested that the presence of microcalcifications in the fibrous cap can contribute to plaque rupture, independently of cap thickness and blood pressure [60] (Figure 2B).

**Figure 3:**
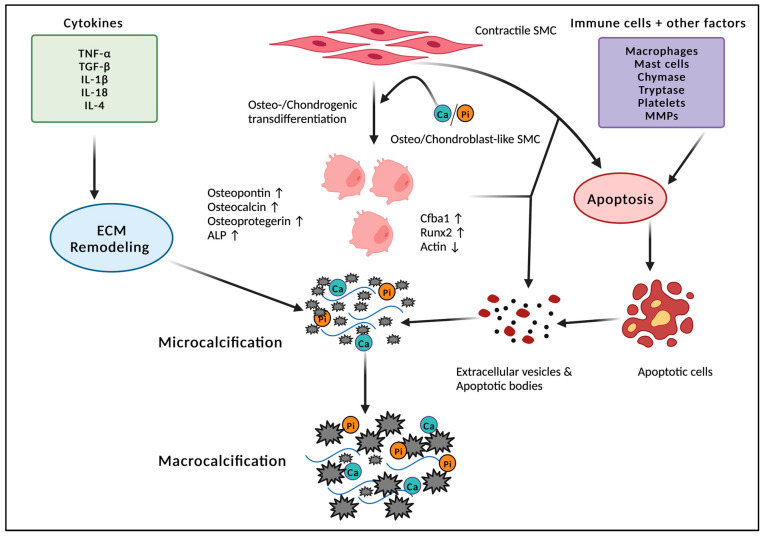
Vascular calcification development. Vascular smooth muscle cells (SMCs) undergo transdifferentiation into an osteo- or chondrogenic phenotype, forming osteo- or chondroblast-like SMCs. Elevated expression of osteo/chondrogenic markers, osteopontin, osteocalcin, osteoprotegerin, ALP, and the osteochondrogenic transcription factors core-binding factor a1 (Cfba1) and Runt-related transcription factor-2 (Runx2) causes the release of extracellular vesicles promoting/inducing calcification. Immune cells, cytokines, and various cytotoxic factors promote calcification by inducing ECM remodeling and apoptosis, causing the formation of extracellular vesicles and apoptotic bodies, which results in microcalcification and macrocalcification of the arteries, causing CAC. This figure was created using BioRender.

Studies on the mechanism for the initiation of CAC suggest that vascular SMCs undergo a phenotypic switch and transdifferentiate to form an osteochondrogenic phenotype [61]. This is accompanied by the expression of the osteochondrogenic markers osteopontin, osteocalcin, osteoprotegerin, ALP/TNAP (alkaline phosphatase/tissue-nonspecific alkaline phosphatase), and the osteochondrogenic transcription factors core-binding factor a1 (Cfba1) and Runt-related transcription factor-2 (Runx2), along with the loss of α-smooth muscle actin [62,63] (Figure 3). It is not known if differentiated vascular SMCs directly switch to osteogenic lineage or first de-differentiate into a mesenchymal-stem-cell-like state before taking a ‘second hit’ that induces re-differentiation into osteogenic lineage [64].

The literature is rife with studies of different factors that affect osteogenic differentiation. Interleukin 18 contributes to osteogenic differentiation in vascular SMCs via TRPM7 (transient receptor potential melastatin 7) channel activation [65] (Figure 3). Interleukin 4 has been shown to regulate osteoprotegerin expression in vascular SMCs and induce calcification [66]. On the other hand, nitric oxide (NO) produced by endothelial nitric oxide synthase (eNOS) prevents vascular SMC osteoblastic differentiation via inhibition of TGF-B signaling [67]. Studies have also shown that dysregulation of eNOS induces oxidative stress, which in turn upregulates Runx2 and promotes vascular SMC calcification [68,69,70].

Despite evidence of the osteogenic transformation of vascular SMCs into osteoblast-like cells, actual bone formation is rarely observed in human coronary arteries [71,72].

Mineral imbalances in the body, such as increased levels of calcium or phosphorous, may promote apatite nucleation and crystal growth in the coronary arteries [72]. Studies have linked high serum phosphate levels to an increased risk of vascular calcification [73]. An increase in phosphate concentration in the vascular SMCs can induce an osteoblast-like phenotype driven by Cfba1/Runx2 (Figure 3). Osteogenic-primed vascular SMCs express ALP and release mineralization-competent extracellular vesicles [74,75]. It has also been suggested that the tumor necrosis factor (TNF-a)–induced nuclear factor-κB promotes inorganic-phosphate-induced calcification of human aortic SMCs [76]. TNF-a and Interleukin 1-b released by macrophages may also induce vascular calcification by contributing to calcium phosphate mineral deposition, SMC apoptosis, and osteogenic differentiation in the plaque (Figure 3). However, the exact mechanism remains elusive [77].

A retrospective cohort analysis of patients has shown a positive correlation between serum IL-1 b levels (>1.8 pg/mL) and high CAC scores [78]. A monoclonal antibody targeting IL-1b, canakinumab, has also been shown to be effective in the primary prevention of atherosclerotic cardiac events, independent of lipid-lowering therapy [79].

The role of NF-kB signaling in atherosclerosis has been well studied, but its role in the development of calcification is complex. Deletion of IKKβ—an essential kinase for NF-κB activation—in vascular smooth muscle cells was shown to promote vascular calcification, mediated by the upregulation of β-catenin and Runx2 and transcription of osteoblast genes. This finding suggests a mechanistic difference between atherosclerosis and calcification [80].

A recent study showed that mannose-receptor-positive macrophages surrounding the calcium deposits in human atherosclerotic plaques cannot resorb calcification, unlike functional osteoclasts, and may potentiate the pro-calcification outcome [81].

Data have shown that there are higher levels of CAC in patients with diabetes mellitus [72]. Upon treatment with advanced glycation end products (AGE), vascular SMCs promote calcification through increased ALP levels and expression of Runx2, suggesting the transformation of vascular SMCs into an osteoblast-like phenotype [82] (Figure 3).

## 3. CAC and Plaque Vulnerability

Atherosclerotic plaque complexity increases with greater degrees of luminal narrowing and the area of calcification [3]. From a clinical point of view, the most critical connection between CAC and plaque is its association with plaque stability [3]. Studies have shown that calcification has a biphasic effect on plaque stability depending on the type and pattern of calcium deposition [37]. The early lesions are composed of spotty calcification, which is associated with a proinflammatory process and increases plaque vulnerability to erosion [38,83] (Figure 2B). Spotty deposition, likely associated with microcalcifications, increases the surface area between calcified and non-calcified areas of the plaque, making it prone to mechanical stress and thus increasing vulnerability to rupture. In fact, percutaneous coronary intervention (PCI) in lesions with spotty calcification is associated with a higher rate of periprocedural myocardial infarction [84] (Figure 2B).

The location of microcalcification and its role in the vulnerability of plaque to rupture are closely linked [85]. Microcalcifications that reside in the fibrous cap tissue affect the biomechanics of the plaque more than those that reside in the necrotic lipid core [60].

Simulations on 3D atherosclerotic plaque models were employed to calculate the vulnerability index, based on the ratio of maximal stresses and ultimate tensile stress in the fibrous cap. The vulnerability index was greatly enhanced by the presence of even a single spherical microcalcification and can potentially transform stable plaques into vulnerable plaques [60].

As calcium deposition progresses and coalesces to become dense macrocalcification, it leads to stabilization of the plaque [38] (Figure 2B). The interface between the calcified and non-calcified areas of the plaque decreases, and thus the plaque becomes more stable [38]; this is mostly observed in fibrotic lesions. A case–control study in patients who experienced acute coronary syndrome (ACS) after having a baseline coronary CT showed that high-density calcified plaques were associated with a lower risk of ACS [86].

The dense and sheet-like matrix calcification is prone to fracture-forming nodular calcification, which may erupt, disrupting the fibrous cap, leading to thrombosis, a life-threatening complication [83]. It has been proposed that nodular calcifications in a coronary artery disrupt the fibrous cap through fragmentation of the necrotic core calcifications, which are flanked by the hard collagen-rich calcifications that are susceptible to mechanical stress [87].

Although calcium burden is heaviest in stable plaques compared with unstable plaques and is inversely related to the necrotic core area, this is not always true. This relationship of CAC to plaque instability seems to differ with age [88]. Subjects in their 4th decade showed higher calcification in unstable lesions than stable lesions, as compared with subjects in their 70s who showed greater calcification in stable plaques, implying that CAC seems to be a determinant of plaque instability in younger individuals.

## 4. Genetic Architecture of CAC

Genetics plays a role in the development of CAC: 40–50% of cases of CAC can be attributed to genetics [89]. Genome-wide association studies have identified several loci linked to CAC, which also appear to be related to CAD [23,90,91].

In 2011, the first GWAS for CAC was conducted, which identified the *9p21* and *6p24* risk loci as being strongly associated with CAC. This finding has been replicated in numerous other GWASs for CAC, as shown by various studies [23,27,28,29,30,31,32,33]. Table 1 summarizes the CAC genome-wide significant loci that have been identified so far in GWASs.

*6p24* is a region that encodes for a phosphatase-1 (PHACTR1) protein, which is involved in the dephosphorylation of multiple substrates and thus regulates cellular processes. GWASs identified three independent SNPs in this locus that are associated with CAC (rs9349379, rs9369640, rs10456561; see Table 1). The exact mechanism through which the *6p24* locus influences CAC is not understood to date. The function of *9p21* is elusive because there is no gene encoding for the protein but a long noncoding RNA of unknown function. The function of the *9p21* locus in CAC is elusive due to the complex and multifaceted nature of the genomic region. There are several SNPs located at the *9p21* that are associated with CAC. Among these SNPs, four are considered CAC GWAS lead SNPs: rs1333049, rs1537370, rs72652478, and rs62555371 (see Table 1). These SNPs are located near the cyclin genes that encode cyclin proteins, which are associated with inflammation and cellular senescence. However, the exact DNA sequence that causes CAC and its connection to these SNPs remain unresolved [23]. The role of *9p21* and *6p24* loci in vascular calcification is currently being investigated [92,93].

Over the last 12 years, there have been significant improvements in genotyping technology, statistical methodologies, and global cooperation, which have led to the identification of 18 CAC risk loci that reach genome-wide significant levels (*p*-value < 5 × 10^−8^). However, to confirm the validity and applicability of the identified risk loci, an increase in the sample size and diversity of the study population in terms of ethnicity and sex is required.

**Table 1:** Summary of genome-wide significant CAC risk loci (October 2023).

Locus ID	Nearest Genes		Index rsIDs	Coordinates (hg38)	GTEx Vascular eQTL		References
	Protein Coding	RNA Coding			Artery	Gene	
1	*APOB*		rs5742904-T	Chr 2:21006288	-	*-*	[33]
2	*PHACTR1, GFOD1, TBC1D7*	*RP1-257A7.4*	rs9349379-A	Chr 6:12903725	Tibial	*PHACTR1, TBC1D7, RP1-257A7.4, GFOD1*	[23,24,29,33]
					Coronary	*PHACTR1*	
					Aorta	*PHACTR1*	
3	*PHACTR1, GFOD1, TBC1D7*	*RP1-257A7.4*	rs9369640-A	Chr 6: 12901209	Tibial	*PHACTR1, RP1-257A7.4*	[23,24,29,33]
					Aorta	*PHACTR1*	
4	*PHACTR1, GFOD1, TBC1D7*	*RP1-257A7.4*	rs10456561-A	Chr 6: 12887233	-	-	[24]
5	*ENPP3, ENPP1*	*miR-548h-5*	rs3844006-C	Chr 6: 131773862	-	-	[24]
6	*IGFBP3*		rs2854746-C	Chr 6: 45921046	Tibial	*IGFBP3*	[24]
					Aorta	*IGFBP3*	
7	*CDKN2A,* *CDKN2B*	*CDKN2B-AS1*	rs1333049-C	Chr 9:22125504	-	-	[23,24,33]
8	*CDKN2A,* *CDKN2B*	*CDKN2B-AS1*	rs1537370-T	Chr 9:22084311	-	-	[24,28,29,33]
9	*CDKN2A,* *CDKN2B*	*CDKN2B-AS1*	rs72652478-C	Chr 9:22102044	-	-	[24]
10	*CDKN2A,* *CDKN2B*	*CDKN2B-AS1*	rs62555371-A	Chr 9: 22107239	-	-	[24]
11	*CXCL12*	*AL512640.1*	rs10899970-A	Chr 10: 44020268	-	-	[24]
12	*ARID5B*		rs9633535-T	Chr 10: 62076329	-	-	[24]
13	*ADK*		rs10762577-A	Chr 10: 74157673	Tibial	*ADK*	[24]
					Coronary	*BMS1P4*	
14	*FGF23*		rs11063120-A	Chr 12: 4377452	-	-	[24]
15	*COL4A1, COL4A2*		rs9515203-T	Chr 13: 110397276	-	-	[24]
16	*CSNK1A1L*	*LINC01048,* *RPS12P24*	rs8000449-T	Chr 13:37224221	-	-	[32]
17	*ADAMTS7*		rs7182103-T	Chr 15: 78831604	Tibial	*ADAMTS7,* *MORF4L1,* *ADAMTS7P3*	[24]
					Coronary	*ADAMTS7*	
					Aorta	*ADAMTS7,* *MORF4L1,* *ADAMTS7P3,* *RPL21P116*	
18	*APOE*		rs7412-T	Chr 19:44908822	-	-	[24,33]

## 5. Race/Ethnicity and Sex in CAC

CAC is a strong predictor of future cardiac events compared with cardiovascular disease (CVD) risk factors and equations in White, African American, Hispanic, and Chinese individuals [3,16]. Racial differences are observed in the degree of CAC, which is an important consideration for clinical outcomes, and thus it is important to understand whether genetics or socioeconomic factors underlie these race-based differences [24]. African Americans carry an adverse cardiovascular profile, but despite that, they show significantly less CAC in asymptomatic individuals [94]. The MESA (Multi-Ethnic Study of Atherosclerosis) study showed that CAC was greatest in White, followed by Chinese, Hispanic, and African American individuals [36]. Histological studies of sudden coronary death victims have also shown that the CAC score was higher in White individuals than in African Americans for every decade till the 70s [30,94,95]. Also, the extent of CAC in explanted hearts follows the same racial pattern for every decade [24,96]. Various possible explanations have been given for the observed racial variation in CAC, e.g., bone mineral density and bone turnover. African Americans have higher bone mineral density and lower bone turnover, possibly accounting for lower arterial calcification [97]. 

South Asians are reported to have higher atherosclerotic cardiovascular disease event rates compared with other ethnicities [97,98], which correlates with the CAC burden [13]. CAC progression is reported to be higher in South Asian men than Chinese, Black, and Latino men, but no difference was observed between South Asian and White men [99,100]. Other reports also show a higher CAC burden in Asian Indians than in other ethnic and racial groups [100,101].

Microarray gene expression profiling of peripheral blood leukocytes from healthy women in the MESA cohort showed that 409 genes were differentially expressed between African American and White individuals with low CAC scores, and 316 genes were differentially expressed between high- and low-CAC-score groups [24]. The results showed that genes characterized by lower expression in African Americans also showed a similar lower expression in individuals with low CAC scores [24]. In another study, the Arg287Gln polymorphism of the soluble epoxide hydroxylase gene significantly predicted CAC in African American but not White individuals; the molecular mechanisms underlying these racial differences remain unclear [102].

Additionally, Wojczynski and colleagues found that the *9p21* locus did not achieve genome-wide significance in African American participants [27]. In the same line, Natarajan and colleagues found that the *9p21* and *6p24* loci (harboring the *PHACTR1* gene) are not significant in African American individuals [33]. Pechlivanis et al. discovered that the chromosome *9p21* SNPs had a more significant effect on the association with CAC in males than females. Conversely, the association effect for rs9349379 in the *6p24* locus with CAC was stronger in females than males [29].

It is a well-known fact that the development of atherosclerosis is affected by sex. The extent of CAC is greater in males up to the 6th decade and becomes equal in the 7th decade of life for females, which suggests that coronary calcification develops rapidly in postmenopausal women [1]. Females develop atherosclerotic CAD 10–15 years later than males, likely due to the protective effect of estrogens in the premenopausal age [34], and it was shown in the Women’s Health Initiative study that females aged 50 to 59 years who were randomized into estrogen therapy groups showed a significantly lower mean CAC score than the placebo [35]. In fact, the CAC burden was three times greater in postmenopausal women than in premenopausal women [35]. The genetic underpinnings of these sex differences in CAC scores are still uncertain, but in women with a low CVD risk, CAC, in addition to traditional risk factors, is strongly related to the incidence of CAD and total mortality [103].

## 6. Association between the Gut Microbiome and CAC

The gut microbiome has emerged as a critical modulator of various physiological processes, including its potential role in CAC development. Studies have shown that specific gut microbial compositions are associated with systemic inflammation and oxidative stress, both of which are key contributors to the pathogenesis of CAC [39,40,104]. Furthermore, gut microbial metabolites, such as trimethylamine-N-oxide (TMAO), have promoted atherosclerosis and vascular calcification by influencing lipid metabolism and arterial inflammation [105,106]. A study has shown that TMAO upregulates the expression of bone-associated molecules like Runx2 and BMP2, indicating that it promotes osteogenic differentiation of vascular smooth muscle cells [107]. Additionally, TMAO positively regulates vascular calcification by activating the NLRP3 inflammasome and NF-κB signaling pathways [107]. It has also been found that TMAO promotes lipogenic differentiation, inhibits osteogenic differentiation of bone marrow mesenchymal stem cells, and increases proinflammatory cytokine production and ROS release [108]. Furthermore, osteogenic endothelial progenitor cells contribute to the endothelial repair of the damage and promote CAD and vascular calcification [109]. However, the exact mechanism remains to be fully elucidated since the available investigations have only demonstrated a correlation between TMAO levels and endovascular calcification. A recent cross-sectional study in Japanese men that investigated the gut microbiota associated with CAC and CAD revealed that the composition of the gut microbiota was significantly different across taxonomic levels, from phylum to genus, and these were dependent on CAC scores, even before the onset of CAD [110]. This may open doors to finding novel prognostic gut biomarkers associated with the development of CAD and novel tools to track CAC progression [110].

Modulating the gut microbiome through dietary interventions or probiotics may offer a novel approach to reducing cardiovascular risk factors and mitigating CAC progression [111]. However, while the evidence suggests a link between the gut microbiome and CAC, further research is needed to fully understand the underlying mechanisms and explore the potential of gut-microbiome-based interventions to prevent and treat CAC.

## 7. Therapeutic Implications in CAC

3-hydroxy-3-methylglutaryl (HMG) CoA reductase inhibitors (statins) are the mainstay of treatment for lowering lipid levels and preventing atherosclerosis, thereby reducing cardiovascular risk in individuals [112,113,114,115,116,117]. These ‘pleiotropic’ effects of statins are thought to be associated with prevention of CAC. However, recent data indicate that statins increase atherosclerotic calcification [118,119,120]. Meta-analyses of clinical trials examining the impact of statin therapy on CAC indicate that statins may expedite the progression of CAC [121].

This paradoxical effect of statin is not completely understood. Statins are postulated to alter the progression and microarchitecture of the calcium deposits by altering their size distribution and density. This change may lead to the coalescence of these calcium deposits, a reduced mineral surface area, and potentially contributing to enhanced plaque stability and ultimately diminishing the risk of plaque rupture [120,121]. The mechanism proposed for the statin-induced calcification is through increased plaque alkaline phosphatase activity and macrophage Rac (Ras-related C3 botulinum toxin substrate)-IL-1β (interleukin-1 beta) signaling axis [122,123].

Guidelines recommend selective use of CAC scoring in decisions to initiate statin therapy in patients with an intermediate risk of CAD, citing studies that show that while the atheroma volume progressed slower in statin users, the calcified atheroma volume increased, indicating that statins may increase the rate of CAC progression [124,125]. The prognostic significance, therefore, is slightly less in statin users [126], which emphasizes a cautious interpretation of CAC scores in statin-treated patients while using CAC data for making clinical decisions about statin treatment intensity [126].

New therapies targeting inflammation and oxidative stress pathways have shown therapeutic potential for CAC. Quercetin has been shown to reduce vascular calcification in animal models by reducing oxidative stress [127]. Studies have explored the use of drugs like canakinumab and antioxidants such as vitamin K in mitigating CAC progression and its associated adverse outcomes [128,129]. Preclinical data on other targeted therapies, including inhibitors of the bone morphogenetic protein pathway and osteoclastic bone resorption, offer promise for future clinical translation [130,131]. Metformin has been shown to be associated with reduced CAC in animal and human studies [132,133,134] evidenced by reduced circulating markers of vascular calcification e.g., osteoprotegerin [135].

While pharmacological approaches have been essential for managing CAC, there is increasing interest in developing precision medicine strategies. Gene therapy targeting specific genetic risk factors associated with CAC holds immense promise for personalized treatment. Research in this area has demonstrated the potential of CRISPR-Cas9-based gene editing to modify genetic variants linked to CAC and atherosclerosis [136,137,138].

Furthermore, advancing knowledge of polygenic risk scores [139,140,141] may enable more accurate risk prediction and facilitate individualized therapeutic approaches. Collectively, these therapeutic strategies and drugs offer hope in mitigating the burden of CAC and reducing cardiovascular morbidity and mortality.

## 8. Present Limitations and Future Directions

GWASs have provided strong evidence that the genetics of CAD and CAC go hand in hand [142]. However, since CAD and the progression of atherosclerotic plaque are closely related to CAC, the genetic associations found could be due to genes/SNPs associated with atherosclerosis rather than those specifically controlling CAC. Additionally, there is a challenge in differentiating the genetic processes involved in intimal versus medial calcifications, which should be examined separately based on solid experimental and clinical evidence.

Human atherosclerotic lesions are unique due to the presence of calcification in the earliest lesions, evidenced by pathological intimal thickening. Although atherosclerotic apolipoprotein E or low-density lipoprotein receptor knockout mice are used to study the development of intimal calcification [143,144], a general limitation is that in humans the longer timespan of decades required for the progression of CAC has to be limited for a study period of several weeks in animal models, and the resultant use of stimuli for the induction of calcification might hamper the comparability [145,146].

Clinically, a CAC score also has limitations. Studies show that physically fit individuals, including athletes with low cardiovascular morbidity and mortality, show higher levels of CAC than age-matched controls after a decade of follow-up [147]. This suggests that the plaque burden may have limitations in risk-stratifying patients [147]. An interesting mechanism presented by Hsu and colleagues suggests that the overall burden of plaque depends on the Western diet and the pattern and location of plaque development, i.e., dense plaques or spotty microcalcification is shaped by physical activity [148].

Vascular calcification is pivotal in cardiovascular disease. Exciting future directions include exploring genetic factors through GWAS, studying epigenetic modifications, investigating gut microbiome interactions, advancing to more sensitive imaging technologies to detect microcalcification and predict plaque rupture, and exploring regenerative medicine interventions.

Recent studies have shown that microcalcification can be detected non-invasively by using molecular imaging such as coronary positron emission tomography (PET) with ^18^F-sodium fluoride—^18^F-NaF [149,150]. ^18^F-NaF binds to microcalcifications owing to the preferential adsorption of fluoride to areas of microcalcification, making it a valuable marker of calcification activity and atherosclerotic plaque vulnerability to rupture [151,152]. Although not clinically implemented yet, it can be employed in targeted management of selected groups of patients with a high risk of adverse events [150].

Integrating these approaches can lead to innovative strategies for managing CAC. From a patient’s point of view, the possibility of a set of genetic variants describing the variations in CAC has the potential to replace currently used CT scans as a safer and improved way of classifying CAD risk.

## 9. Conclusions

In conclusion, the role of CAC in the development of CAD and future cardiovascular events is of utmost importance. As we gain more insights into the genetic underpinnings and molecular mechanisms of CAC, we may be able to develop personalized prevention and treatment strategies. A comprehensive understanding of CAC is essential for improving clinical outcomes and reducing the burden of cardiovascular disease. Future studies should include more diverse populations and a balanced sex cohort to identify genetic variants specific to ethnic groups or sex.
